# Flavonoid glycoside fraction of *Ginkgo biloba* extract modulates antioxidants imbalance in vanadium-induced brain damage

**DOI:** 10.3934/Neuroscience.2023015

**Published:** 2023-06-29

**Authors:** Adeshina O. Adekeye, Adedamola A. Fafure, Morayo M. Omodele, Lawrence D. Adedayo, Victor O. Ekundina, Damilare A. Adekomi, Ephraim Samuel Jen, Thomas K. Adenowo

**Affiliations:** 1 Department of Anatomy, College of Medicine and Health Sciences, Afe Babalola University, Ado-Ekiti, Ekiti State, Nigeria; 2 Department of Physiology, College of Health Sciences, Bowen University, Iwo, Osun State, Nigeria; 3 Department of Medical Laboratory Science, College of Medicine and Health Sciences, Afe Babalola University, Ado-Ekiti, Ekiti State, Nigeria; 4 Department of Anatomy, Faculty of Basic Medical Sciences, College of Health Sciences, Osun State University, Osogbo, Nigeria; 5 Department of Anatomy, Faculty of Basic and applied Sciences, Lead City University, Ibadan, Oyo State, Nigeria

**Keywords:** ginkgo biloba extract, inflammasome, vanadium, NeuN, Glutathione peroxidase

## Abstract

Human and animal diseases have always been reported to be treated by medicinal herbs owing to their constituents. Excess sodium metavanadate is a potential environmental toxin when consumed and could induce oxidative damage leading to various neurological disorders and Parkinsons-like diseases. This study is designed to investigate the impact of the flavonoid Glycoside Fraction of Ginkgo Biloba Extract (GBE) (at 30 mg/kg body weight) on vanadium-treated rats. Animals were divided randomly into four groups: Control (Ctrl, normal saline), Ginkgo Biloba (GIBI, 30mg/kg BWT), Vanadium (VANA, 10 mg/kg BWT) and Vanadium + Ginkgo biloba (VANA + GIBI). Markers of oxidative stress (Glutathione Peroxidase and Catalase) were assessed and found to be statistically increased with GIBI when compared with CTRL and treatment groups. Results from routine staining revealed that the control and GIBI group had a normal distribution of cells and a pronounced increase in cell count respectively compared to the VANA group. When compared to the VANA group, the NeuN photomicrographs revealed that the levels of GIBI were within the normal range (***p < 0.001; ** p < 001). The treatment with GIBI showed a better response by increasing the neuronal cells in the VANA+GIBI when compared with the VANA group. The NLRP3 Inflammasome photomicrographs denoted that there was a decrease in NLRP3-positive cells in the control and GIBI groups. The treatment group shows fewer cells compared to that of the VANA group. The treatment group shows fewer cells compared to that of the VANA group. The findings of the study confirmed that ginkgo biloba extract via its flavonoid glycoside fraction has favorable impacts in modulating vanadium-induced brain damage with the potential ability to lower antioxidant levels and reduce neuroinflammation.

## 1. Introduction

Parkinson's disease is a complicated neurodegenerative disorder where the risk, onset, and development of the illness are influenced by both uncommon and common genetic variations [1],[2]. Parkinson's disease (PD) is known to be a progressive neurological movement condition that affects 1% of people above the age of 55 and rises 5% by the age of 85 [3],[4]. It is also a neurodegenerative disorder characterized by the progressive loss of dopaminergic neurons in the substantia nigra, a region of the brain responsible for motor control. Parkinson's disease is typically brought on by nerve cell death in the caudate putamen, substantia nigra, midbrain, and globus pallidus region of the brain. Parkinson's disease symptoms and progression rates differ from person to person. The common symptoms include Bradykinesia, tremors at rest, rigidity, and postural instability [5]. The exact causes of PD are not fully understood, but there is growing evidence suggesting that environmental factors can play a role in its development. One of the environmental factors is exposure to metals, including manganese, lead, cadmium, and vanadium [6].

Vanadium (V) is a chemical element that is a silvery-white soft metal in Periodic Table Group 5. High-speed tool steel, high-strength low-alloy steel and wear-resistant cast iron, it is alloyed with steel and iron [7]. One of the crucial trace elements for both humans and animals is Vanadium (V) and participates in many important physiological activities, such as promoting hematopoietic function, reducing blood glucose concentration, protecting the islet cell, lessening atherosclerosis, and performing anti-hyperlipidemic, anti-hypertension or anti-apoptosis functions [8],[2]. The Spanish mineralogist Andrés Manuel del Ro discovered vanadium in 1801 and termed it erythronium, but later realized it was just impure chromium. In the earth's crust, Vanadium ranks as the 22nd most common element and it is present in a variety of minerals, and petroleum. Through a wide variety of roasting, smelting, and leaching processes, vanadium pentoxide (V_2_O_5_) is extracted from ores [9]. During the production of sulfuric acid, vanadium compounds (pentoxide and certain vanadates) have been used as catalysts. Vanadium occurs in nature as two isotopes: of vanadium in nature: stable vanadium-51 (99.76%) and radioactive vanadium-50 which is weak (0.24%). Heavy metals like vanadium persist for a very long time in the environment and so severely affect human health. The chemical industry makes substantial use of vanadium, which is also released into the atmosphere when fossil fuels are burned through forest fires, volcanic emissions, and marine aerosols. Vanadium has been shown to pass the blood-brain barrier and its experiments on laboratory animals have been linked to demyelination, microglia and astrocyte activation, tumor necrosis factor and interleukin 1β expression, and locomotion and cognition deficits. Antioxidants are chemical compounds which bind to free oxygen radicals and prevent these radicals from damaging healthy cells [10]. Vanadium, when consumed in high concentrations (greater than 1.8 mg per day) may cause liver or kidney damage, other studies link elevated blood vanadium levels to a higher risk of breast cancer [11]. It has the ability to produce reactive oxygen species (ROS) and induces oxidative stress, which can result in neuronal cell damage and apoptosis. Vanadium has also been shown to down-regulate mitochondrial function, impair protein degradation mechanisms, and cause inflammation, all of which are implicated in the pathogenesis of Parkinson's disease. Understanding the connections between vanadium, oxidative stress, inflammation, and the progression of Parkinson's disease is crucial for developing therapeutic strategies and interventions to mitigate the impact of environmental factors. Ginkgo Biloba is a species of tree native to China. It is regarded as a “living fossil” because of the species' uninterrupted existence for 270 million years without changes and for being the oldest tree in the world, with no living relative in existence and is the last living species of the Ginkgoales [12]. Ginkgo contains potent antioxidants, which fight the damaging effects of free radicals, and has anti-inflammatory potential, which might be the reason behind most of its health claims [13]. Thus, our study investigates the impact of the flavonoid Glycoside Fraction of Ginkgo Biloba on vanadium induced neurotoxicity.

## 2. Materials and methods

### 2.1. Materials

The following supplies were utilized in the experiment; 40 adult male Wistar rats, plastic cages with metal covers to keep the animals together, water bottles to hydrate the rats, beddings composed of wood shavings and sawdust and the animals were fed feed. NLRP3 inflammasome, 2-step plus poly-HRP anti mouse/rabbit IgG detection system, DAB solution, phosphate buffer solution (PBS), triton X, RBFOX3 polyclonal antibody (Elabscience BiotechnologyInc; Wuhan, Hubei, P.R.C., China).

### 2.2. Animal care and management

Male Wistar rats (40, *Rattus Norvegieus*) each weighed between 160–230 g were bought from the animal house holding unit of Afe Babalola University, Ado Ekiti. The animals were acclimatized in Afe Babalola University's animal house, under standard laboratory conditions, and fed with pelletized mouse feed (ABUAD Feeds, Ekiti State, Nigeria) and water ad libitum. The uses and care of animal procedures in this research were in compliance with the Research Ethics Committee of the College of Medicine and Health Sciences, Afe Babalola University, Ado-Ekiti, Nigeria (ABUAD/ERC/016/2022), National Research Centre ethics committee guidelines and the National Institutes of Health's Guide for the Care and Use of Laboratory Animals (Publication no. 19–60, revised 1985).

### 2.3. Treatment

Control group received vehicle (Distilled water) by oral gavage. GIBI group received 32 mg/kg of Gingko biloba orally. VANA group received 10mg/kg of vanadium intraperitoneally. GIBI and VANA group received 10 mg/kg of vanadium followed by 32 mg/kg of Gingko *biloba* as a post treatment [2],[14]. The administration lasted for 3 weeks.

### 2.4. Animal sacrifice

The animals were anesthetized using intraperitoneal injection of 50 mg/kg b.w. of sodium pentobarbital, followed by sacrificed through intracardiac perfusion fixation with phosphate buffer solution and 10% formal saline respectively.

### 2.5. Brain sample collection and preservation

The brain sample was collected after the sacrifice. The region of the globus pallidus was grossed. The samples were homogenized in phosphate buffer solution and centrifuged at 10,000 rpm for 10 min at 4 °C. The supernatant was decanted and stored for spectrophotometric analysis for Glutathione peroxidase and catalase activity using commercial kits. The samples for histological and immunohistochemical analysis were post-fixed in 10% formal saline following intracardiac perfusion fixation.

### 2.6. Estimation of Glutathione peroxidase (GPx) and Catalase

GPx activity was assessed using the method of the Paglia et al. approach [15]. The following substances were placed in a tube to begin the enzymatic reaction: A spectrophotometer was used to measure the levels of Nicotinamide Adenine Dinucleotide Phosphate (NADPH), reduced glutathione (GSH), sodium azide, and glutathione reductase after the injection of H2O2 and absorbance changes were monitored at 340 nm. Units per gram of protein were used to measure the activity.

The level of Catalase activity was measured spectrophotometrically by utilizing the method of Koroliuk et al. [16]. In a nutshell, 10 *µ*L of the sample was treated for 10 min with 100 *µ*mol/mL of H_2_O_2_ in 0.05 mmol/L Tris-HCl buffer with pH of 7. A quick addition of 50 *µ*L of 4% ammonium molybdate stopped the reaction. At 410 nm the yellow combination of ammonium molybdate and H_2_O_2_ was detected. the quantity of enzymes needed to decompose 1 *µ*mol of H2O2 per min was used to define one unit of catalase activity. The method of Benzie and Strain [17] was used to measure the serum's total antioxidant capacity. By combining buffer acetate TPTZ solution in HCl, FRAP (ferric reducing antioxidant power) was created as a functional solution. FeCl_3_ was then mixed in and added after that 8 *µ*L of serum and 240 *µ*L of the aforementioned working solution were combined and incubated at room temperature for 10 min. At 532 nm, the optical densities of the samples were determined. As a measure of total antioxidant capacity was expressed as mmol/L was used.

### 2.7. Immunohistochemical Analysis

Following routine tissue processing, sections of the globus pallidus (5u thick) were coronally cut on a microtome and immunolabelled with primary antibodies directed against RBFOX3 (for neuronal cell distribution) and NLRP3 polyclonal antibodies (for inflammation) (NLRP3 1:200; RBFOX3 1:150, Elabscience, China).

### 2.8. Tissue Photomicrography

The image was captured and analyzed using an OPTO-Edu industrial camera light microscope connected to a computer with an image-processing and analysis software Image-J (Version 1.53). The total positive cells in the globus pallidus were counted and calculated in a given square area. 30 sections (5 µm) of the globus pallidus in each animal were analyzed [18].

### 2.9. Statistical Analysis

One-way ANOVA was used to evaluate the data, and then the student Newman-Keuls (SNK) test for multiple comparisons. The statistical package used for data analysis was Graph Pad Prism 5 (Version 5.03, Graphpad Inc.) and the significant difference was set at p < 0.05.

## 3. Results

### 3.1. Glutathione Peroxidase (GPx)

**Figure 1.**
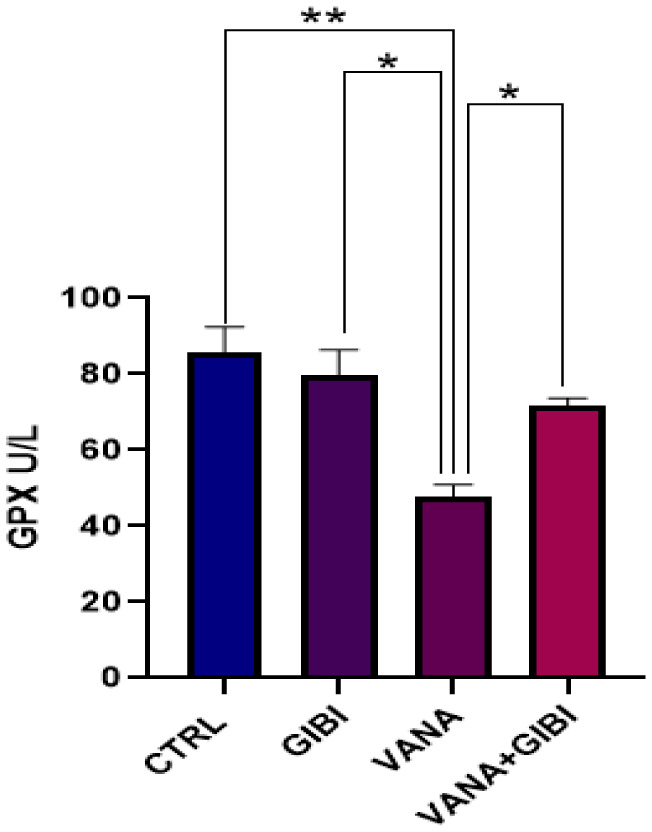
Graph representing the concentration of GPX (U/L) in the brain tissue of the experimental animal. Control and GIBI group revealed a statistically significant increase in GPX level when compared to the vanadium group (**p < 0.01; *p < 0.05). GIBI was seen to statistically increased the level of GPX in the VANA+GIBI group in comparison with the vanadium group only (*p < 0.05). *CTRL = Control, GIBI = Ginkgo biloba, VANA = Vanadium, VANA + GIBI = Vanadium + Ginkgo biloba*.

### 3.2. Catalase (CAT)

**Figure 2.**
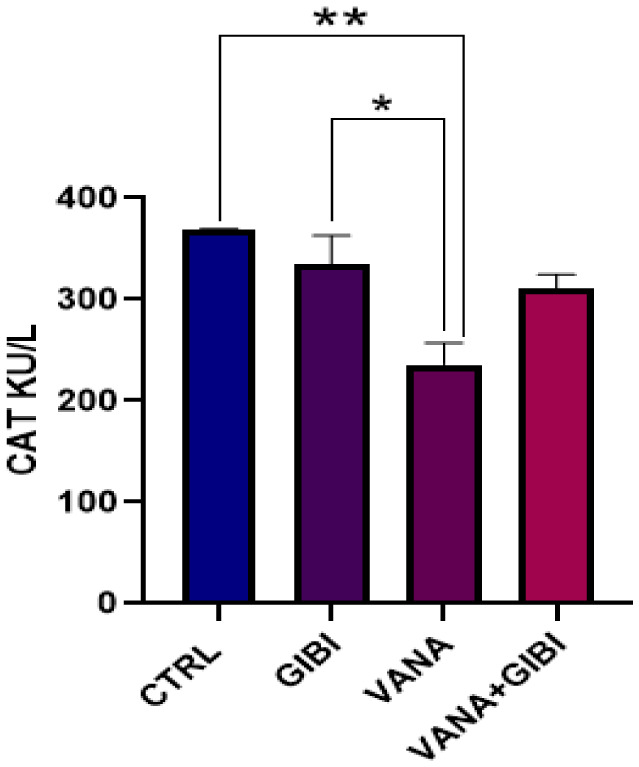
Graph representing the concentration of CAT (KU/L) in the brain tissue of the experimental animals. Control and GIBI group revealed a significant increase in the level of catalase concentration when compared to the vanadium group (**p < 0.01; *p < 0.05). A rise in the level of catalase concentration was noticed in the VANA+GIBI group in comparison with the vanadium group only. *CTRL = Control, GIBI = Ginkgo biloba, VANA = Vanadium, VANA + GIBI = Vanadium + Ginkgo biloba*.

### 3.3. Histological Analysis

**Figure 3.**
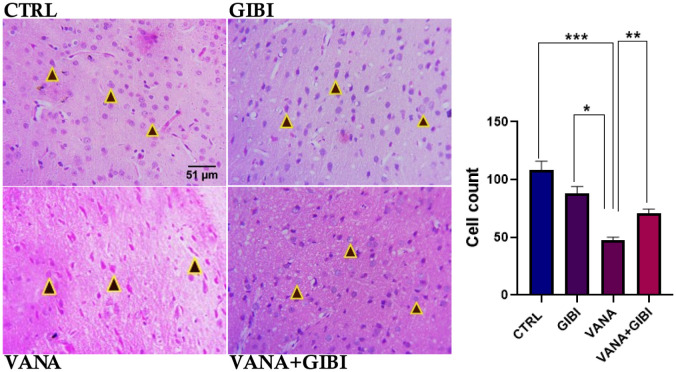
Photomicrograph showing the globus pallidus histoarchitecture in the vanadium induced experimental animals. Control, GIBI revealed normal and even distribution of cells as well as a significant increase in cell count when compared to the vanadium group (***p < 0.001; *p < 0.05). VANA+GIBI group shows the recovery of globus pallidus cells and cells count significant rise in comparison to the vanadium group (**p < 0.01). *CTRL = Control, GIBI = Ginkgo biloba, VANA = Vanadium, VANA + GIBI = Vanadium + Ginkgo biloba*. The brown arrow represents the Globus Pallidus (GP) cells. Stained with H&E (Mag. x1200).

### 3.4. Immunohistochemistry

These results demonstrated the neuronal cell distribution as well as the inflammatory process in the vanadium induced experimental animals using NeuN and NLRP3 markers respectively.

**Figure 4.**
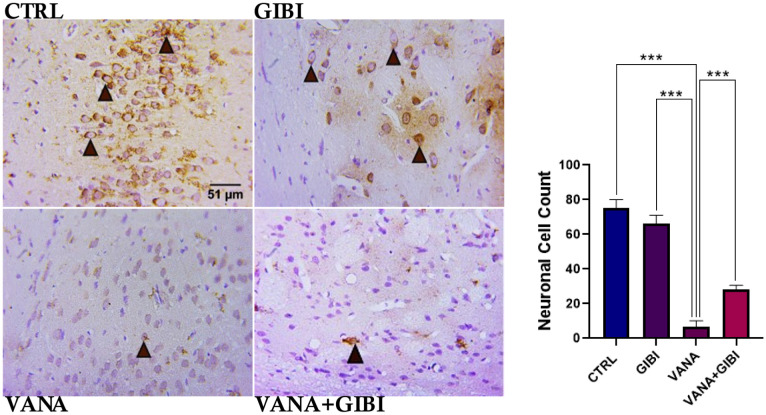
Immunohistochemical photomicrograph showing the neuronal cell distribution pattern and neuronal cell count of the globus pallidus in the vanadium induced experimental animals. Control, GIBI revealed normal neuronal cells as well as significant increase in cell count when compared to the vanadium group (***p < 0.001; ***p < 0.001). Treatment with GIBI shows an ameliorative response by significantly increasing the neuronal cell in the VANA+GIBI group when compared to the vanadium group (***p < 0.001). *CTRL = Control, GIBI = Ginkgo biloba, VANA = Vanadium, VANA + GIBI = Vanadium + Ginkgo biloba*. Brown arrow and dark brown cells indicate the immune-positive cells. Stained with H&E (Mag. x1200).

### 3.5. NLRPL3 Inflammasome

**Figure 5.**
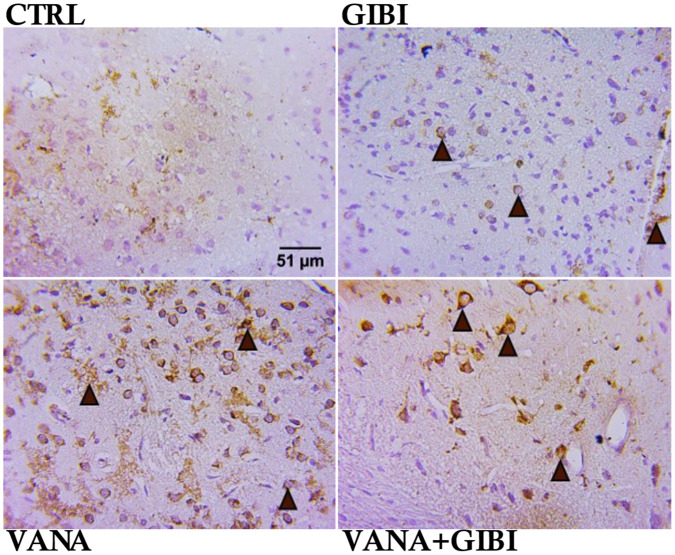
Immunohistochemical photomicrograph showing the NLRP3 inflammasome expression in the vanadium induced experimental animals. Control, GIBI revealed little or no expression as well as a significant reduction in NLRP3 immuno-reactive cells when compared to the vanadium group (***p < 0.001; ***p < 0.001). Treatment with GIBI was effective by the significant reduction in the NLRP3 inflammasome expression in the VANA+GIBI group in comparison with the vanadium group (***p < 0.001). *CTRL = Control, GIBI = Ginkgo biloba, VANA = Vanadium, VANA + GIBI = Vanadium + Ginkgo biloba*. Brown arrow and dark brown cells indicate the NLRP3-positive cells.Stained with H&E (Mag. x1200).

## 4. Discussion

Vanadium toxicity has emerged as a significant health concern due to the widespread industrial use and the potential adverse effects on human health and the environment. In lieu of these, an ameliorative response is crucial to protect human health against vanadium neurotoxicity. Increased amounts of reactive oxygen species (ROS) and inadequate activity of antioxidant systems constitute oxidative stress [19]. The term “Glutathione Peroxidase” (GPx) refers to a class of peroxidase active enzymes whose primary biological function is to shield the body's organs against oxidative damage. Glutathione peroxidase converts free hydrogen peroxide into water and lipid hydroperoxides into appropriate alcohols in its biochemistry [20]. In this study, it was seen that GIBI was seen to statistically increased the level of GPx in the VANA+GIBI group when compared with the vanadium group only (*p < 0.05) (Figure 1), and this increase indicates the possible involvement of GIBI in enhancing the antioxidant capacity of the induced animals. Glutathione peroxidase is a critical enzyme that utilizes glutathione to neutralize harmful reactive oxygen species (ROS). The elevation of GPx levels suggests that GIBI could enhance the activity of this enzyme, thereby promoting the detoxification of ROS induced by vanadium. In addition, a rise in the level of catalase concentration was noticed in the VANA+GIBI group when compared with the vanadium group only, but not statistically significant (Figure 2). Similarly, the rise in catalase concentration in the VANA+GIBI group suggests that GIBI may stimulate the synthesis or activity of catalase, which plays a crucial role in breaking down hydrogen peroxide, a harmful ROS, into water and oxygen. This elevation in catalase levels indicates that GIBI may augment the defense mechanisms against vanadium-induced oxidative stress. It has been shown that vanadium-induced pulmonary inflammation, neurotoxicity, and carcinogenic-related effects are exacerbated by ROS and oxidative stress [2],[21].

NeuN is a neuronal nuclear antigen that is commonly used as a biomarker for neurons [22]. The present study indicates the increase in neuronal cell distribution pattern in the control and GIBI treated groups, while a disruption in the neuronal architecture and significant reduction in the neuronal count was observed in the Vanadium treated group. Treatment with GIBI shows an ameliorative response by significantly increasing the neuronal cell in the VANA+GIBI group when compared to the vanadium group (***p < 0.001) (Figure 4). According to Adekeye et al. [2], the reduction in the neuronal level might be due to the vanadium's toxic properties which might affect the brain leading to neuroinflammation, and memory deficits which are supported according by Olanrewaju et al. [23].

Inflammasomes are cytosolic multiprotein oligomers of the innate immune system that oversee initiating inflammatory reactions [24]. Neuroinflammation has been implicated in the pathogenesis of several neurological disorders, including Alzheimer's disease, Parkinson's disease, and Parkinson-like diseases [25],[26]. The inflammatory response in the brain exacerbates the underlying damage, leading to neuronal damage or degeneration in these disorders. This study revealed the ameliorative role of Gingko biloba as seen in the GIBI group which shows little or no inflammatory cells or a significant reduction in NLRP3 immuno-reactive cells when compared to the vanadium group (***p < 0.001) (Figure 5). Treatment with GIBI was effective by significantly reducing the NLRP3 inflammasome expression in the VANA+GIBI group when compared with the vanadium-treated group (***p < 0.001). These findings suggest that Gingko biloba treatment can effectively reduce the presence of inflammatory cells by inhibiting the expression of NLRP3 inflammasome, indicating its potential as a therapeutic intervention for conditions associated with inflammation. The histological analysis revealed a normal and even distribution of the Globus Pallidus (GP) cells in the control and GIBI groups, while there is a statistically significant increase in the VANA+GIBI group when compared with the VANA group (**p < 0.01) (Figure 3). As a free radical scavenger, *Ginkgo biloba* extract shields neurons against oxidative damage and apoptosis brought on by aging, cerebral ischemia, and neurodegenerative disorders. *Ginkgo biloba* also inhibits inflammation and protects against hypoxic challenges and increased oxidative stress. Ginkgo Biloba can enhance the activities of antioxidant enzymes, such as superoxide dismutase (SOD), glutathione peroxidase (GSH-Px), and catalase, thereby indirectly contributing as an antioxidant [27].

## 5. Conclusion

The results from the study revealed that ginkgo biloba extract has a favorable impact on vanadium-induced motor deficit via its flavonoid glycoside fraction with a potential ability to reduce neuroinflammation in the globus pallidus.

## References

[1] Blauwendraat C, Nalls MA, Singleton AB (2020). The genetic architecture of Parkinson's disease. Lancet Neurol.

[2] Adekeye AO, Irawo-Joseph G, Fafure AA (2020). Ficus exasperate vahl leaves extract attenuates motor deficit in vanadium-induced parkinsonism mice. Anat Cell Biol.

[3] Adekeye AO, Adefule AK, Shallie P (2018). Cholecalciferol Attenuates 1-Methyl-4-Phenyl-1,2,3,6-Tetrahydropyridine-Induced Parkinson's Like-Disease Variation in Oxidative Stress Markers, Mouse Behaviour and Cellular morphology of Striatum and Substantia nigra. Anat J Africa.

[4] Berkovits M, Farrar J (2010). Conflicting emotions: The connection between affective perspective taking and theory of mind. Brit J Dev Psyschol.

[5] Han W, Ahn D, Kim S (2018). Psychiatric Manifestation in Patients with Parkinson's Disease. J Korean MedSci.

[6] Simon DK, Tanner CM, Brundin P (2020). ParkinsonDiseaseEpidemiology, Pathology, Genetics and Pathophysiology. Clin Geriatr Med.

[7] Antrekowitsch H, Luidold S, Gaugl H (2021). Thermodynamic Calculations of the Production of Ferrovanadium from V-containing Steelworks Slags with a low Content of V2O5.Part 1.

[8] Li H-Y, Fang H-X, Wang K (2015). Asynchronous extraction of vanadium and chromium from vanadium slag by stepwise sodium roasting–water leaching. Hydrometallurgy.

[9] Zou K, Junhu X, Guanjie L (2021). Effective Extraction of Vanadium from Bauxite-Type Vanadium Ore Using Roasting and Leaching. J Metals.

[10] Tiwari AK (2004). Antioxidants: New generation therapeutic base for treatment of polygenic disorders. Curr Sci.

[11] Tang L, Su Y, He J (2012). Urinary Titanium and Vanadium and Breast Cancer: A Case-Control Study. Nutr Cancer.

[12] Isah T (2015). Rethinking Ginkgo biloba L.: Medicinal uses and conservation. Pharmacognosy Rev.

[13] Mei N, Guo X, Ren Z (2017). Review of Ginkgo biloba-induced toxicity, from experimental studies to human case reports. J Environ Sci Health C Environ Carcinog Ecotoxicol Rev.

[14] Zhang Q, Wang G, Ji-ye A (2009). Application of GC/MS-based metabonomic profiling in studying the lipid-regulating effects of *Ginkgo biloba* extract on diet-induced hyperlipidemia in rats. Acta Pharmacologica Sinica.

[15] Paglia DE, Valentine WN (1967). Studies on the quantitative and qualitative characterization of erythrocyte glutathione peroxidase. J Lab Clin Med.

[16] Koroliuk MA, Ivanova IG, Maiorova (1988). A method of determining catalase activity. LaboratornoeDelo.

[17] Benzie IF, Strain JJ (1996). The ferric reducing ability of plasma (FRAP) as a measure of antioxidant power. FRAP Assay Anal Biochem.

[18] Adekeye AO, Fafure AA, Ogunsemowo AE (2022). Naringin ameliorates motor dysfunction and exerts neuroprotective role against vanadium-induced neurotoxicity. AIMS Neurosci.

[19] Preiser JC (2012). Oxidative Stress. Jpen Parenter Enter.

[20] Muller L, Lustgarten S, Jang Y (2007). Trends in oxidative aging theories. Free Radical Bio Med.

[21] Afeseh Ngwa H, Kanthasamy A, Anantharam V (2009). Vanadium induces dopaminergic neurotoxicity via protein kinase Cdeltadependent oxidative signaling mechanisms: relevance to etiopathogenesis of Parkinson's disease. Toxicol Apply Pharmacol.

[22] Kim J, Basak JM, Holtzman DM (2009). The role of apolipoprotein E in Alzheimer's disease. Neuron.

[23] Fatola OI, Olaolorun FA, Olopade FE (2018). Trends in vanadium neurotoxicity. Brain Res Bull.

[24] Mariathasan S, Newton K, Monack DM (2004). Differential activation of the inflammasome by caspase-1 adaptors ASC and Ipaf. Nature.

[25] Fafure AA, Adekeye AO, Enye LA (2018). Ficus ExasperataVahl improves manganese-induced neurotoxicity and motor dysfunction in mice. Anat J Africa.

[26] Folarin OR, Snyder AM, Peters DG (2017). Brain metal distribution and neuro-inflammatory profiles after chronic vanadium administration and withdrawal in mice. Front Neuroanat.

[27] Brondino N, De Silvestri A, Re S (2013). A Systematic Review and Meta-Analysis of Ginkgo biloba in Neuropsychiatric Disorders: From Ancient Tradition to Modern-Day Medicine. Evid Based Compl Alt.

